# Correction: A planar pentacoordinate oxygen in the experimentally observed [Be_5_O_6_]^2−^ dianion

**DOI:** 10.1039/d5sc90136g

**Published:** 2025-07-02

**Authors:** Rui Sun, Yang Yang, Xin Wu, Hua-Jin Zhai, Caixia Yuan, Yan-Bo Wu

**Affiliations:** a Key Laboratory of Chemical Biology and Molecular Engineering, Ministry of Education, Key Laboratory of Materials for Energy Storage and Conversion of Shanxi Province, Institute of Molecular Science, Shanxi University 92 Wucheng Road Taiyuan Shanxi 030006 People's Republic of China wyb@sxu.edu.cn cxyuan@sxu.edu.cn; b Basic Sciences Department, Shanxi Agricultural University 1 South Mingxian Road Taigu Shanxi 030801 People's Republic of China

## Abstract

Correction for ‘A planar pentacoordinate oxygen in the experimentally observed [Be_5_O_6_]^2−^ dianion’ by Rui Sun *et al.*, *Chem. Sci.*, 2025, https://doi.org/10.1039/d5sc02361k.

The authors regret that the use of the phrase “electrospray ionization” was incorrect in two instances in their published articles. The affected sentences are:

“The [Be_5_O_6_]^2−^ dianion, first produced in 2006 *via* electrospray ionization and initially proposed by a concurrent computational study to adopt a linear O–Be alternating structure, stands as a rare experimentally observed SMCA.”

And

“Notably, a literature survey revealed that the corresponding [Be_5_O_6_]^2−^ dianion was generated in 2006 *via* electrospray ionization,^37^ but a concurrent computational study^38^ incorrectly proposed a linear O–Be-alternating structure (**0** in Fig. 1).”

The phrase “electrospray ionization” is hereby corrected to “simultaneous metal sputtering and O_2_ flooding”. The sentences above are corrected to:

“The [Be_5_O_6_]^2−^ dianion, first produced in 2006 *via* simultaneous metal sputtering and O_2_ flooding and initially proposed by a concurrent computational study to adopt a linear O–Be alternating structure, stands as a rare experimentally observed SMCA.”

And

“Notably, a literature survey revealed that the corresponding [Be_5_O_6_]^2−^ dianion was generated in 2006 *via* simultaneous metal sputtering and O_2_ flooding,^37^ but a concurrent computational study^38^ incorrectly proposed a linear O–Be-alternating structure (**0** in Fig. 1).”

The Royal Society of Chemistry apologises for these errors and any consequent inconvenience to authors and readers.

